# Alternative Lengthening of Telomeres: A Prognostic Paradox in Cancer

**DOI:** 10.3390/cells14201613

**Published:** 2025-10-17

**Authors:** Ji-Yong Sung

**Affiliations:** Department of Neurosurgery, Seoul National University Bundang Hospital, Seongnam-si 13620, Republic of Korea; 5rangepineapple@gmail.com

**Keywords:** alternative lengthening of telomeres (ALT), telomere maintenance mechanisms, ATRX/DAXX mutations, genomic instability, DNA damage response (DDR), glioblastoma (GBM), chondrosarcoma, immune-cold tumors, prognostic biomarker

## Abstract

**Highlights:**

**What are the main findings?**

- ALT functions as a context-dependent double-edged sword: it drives aggressiveness in rapidly proliferating cancers but imposes replicative and metabolic stress in slow-growing, immune-cold tumors such as glioblastoma and chondrosarcoma.
- Loss of ATRX/DAXX and ALT activation creates unique DNA repair vulnerabilities, including hypersensitivity to ATR and PARP inhibition, opening avenues for targeted therapy.

**What is the implication of the main finding?**

- Refines prognostic interpretation of ALT positivity—transforming it from a uniformly negative marker into a tumor-type-specific indicator of clinical outcome.
- Positions ALT as a precision oncology target, where integrated biomarkers (C-circle as say, APB detection, ATRX/DAXX mutation profiling) enable patient stratification and therapeutic decision-making.

**Abstract:**

Telomere maintenance enables unlimited cell proliferation by counteracting telomere erosion. While the majority of tumors activate telomerase, a significant subset—approximately 10–15%—utilizes alternative lengthening of telomeres (ALT), a recombination-based mechanism. ALT-positive cancers are classically associated with genomic instability, anaphase bridges, chromosomal rearrangements, and resistance to DNA-damaging therapies. This process is closely associated with genetic instability, which contributes to chromosomal rearrangements and tumor evolution. Consequently, ALT has traditionally been considered an adverse prognostic marker in aggressive malignancies such as osteosarcoma, pancreatic neuroendocrine tumors, and high-grade sarcomas. Paradoxically, recent evidence demonstrates that ALT positivity correlates with improved survival in glioblastoma (GBM) and chondrosarcoma, two tumor types that have historically been regarded as immune-cold and therapeutically intractable. This favorable outcome likely reflects a convergence of factors, including replication stress and DNA damage that impose a fitness cost in slow-growing or metabolically constrained tumors. Loss of ATRX/DAXX, while enabling ALT, further amplifies chromatin fragility, and ALT-mediated instability may paradoxically enhance immunogenicity within immune-quiescent microenvironments. Moreover, ALT-positive cells exhibit unique therapeutic vulnerabilities, particularly to ATR and PARP inhibitors. Together, these observations support a context-dependent model in which ALT functions as a double-edged sword, acting as a driver of malignant aggressiveness in rapidly proliferating cancers while serving as a relative liability in slower-growing, immune-cold tumors. Understanding this duality not only refines prognostic stratification but also opens opportunities for precision oncology. By integrating ALT-specific biomarkers into clinical workflows and exploiting ALT-related DNA repair dependencies, clinicians may transform a once uniformly negative prognostic factor into an actionable therapeutic target.

## 1. Introduction

Telomeres are nucleoprotein structures composed of repetitive nucleotide sequences and associated proteins collectively known as the shelterin complex, which play a critical role in maintaining genomic stability by preventing end-to-end chromosome fusions, degradation, and inappropriate DNA repair. During normal cellular proliferation, telomeres progressively shorten as a result of both the end-replication problem and oxidative stress, which together accelerate telomere erosion and ultimately trigger cellular senescence or apoptosis once telomeres reach a critically short length. Importantly, telomere shortening primarily occurs in human somatic cells that lack telomerase activity, whereas cells expressing telomerase—such as germline cells, stem cells, lymphocytes, and endothelial cells—can at least partially counteract telomere attrition. To bypass this proliferative barrier, cancer cells must activate a telomere maintenance mechanism (TMM). The most common strategy is reactivation of telomerase, a reverse transcriptase (hTERT) that elongates telomeres by adding TTAGGG repeats. Telomerase activity is detected in ~85–90% of human cancers, underscoring its central role in tumor immortalization. However, a significant minority of cancers [1]—approximately 10–15%—utilize a telomerase-independent mechanism known as alternative lengthening of telomeres (ALT) [2,3]. ALT is predominantly activated in tumors originating from mesenchymal and neuroendocrine tissues, consistent with previous reports [4].

**Figure 1:**
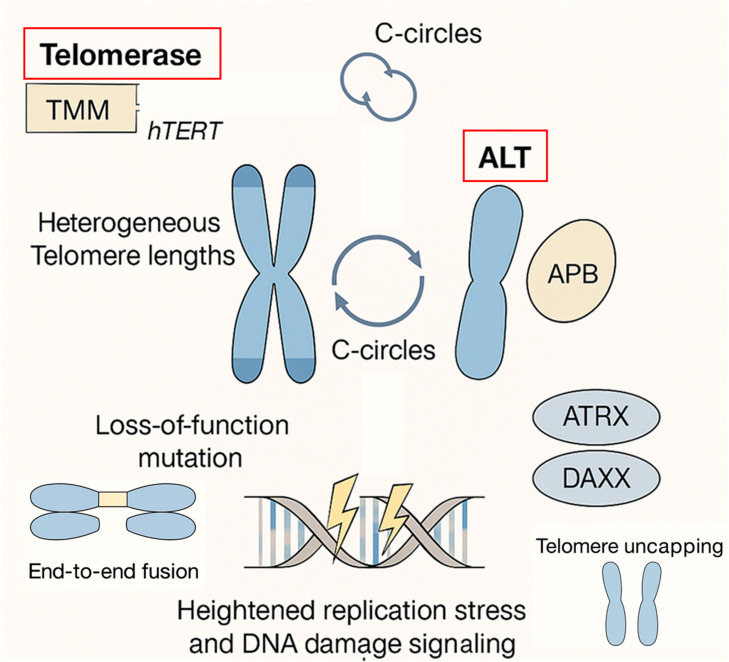
**Mechanistic Overview of Alternative Lengthening of Telomeres (ALT).** This diagram illustrates the distinct molecular features and consequences of ALT as a telomere maintenance mechanism. Unlike telomerase (hTERT)-dependent elongation, ALT operates through homologous recombination, often triggered by loss-of-function mutations in chromatin remodelers such as ATRX and DAXX. ALT-positive cells exhibit heterogeneous telomere lengths, accumulate extrachromosomal telomeric DNA circles (C-circles), and form ALT-associated promyelocytic bodies (APBs), which serve as recombination hubs. The recombination-based telomere synthesis imposes replication stress and activates persistent DNA damage signaling, contributing to genomic instability and tumor-specific vulnerabilities.

ALT is based on homologous recombination-mediated DNA synthesis, in which telomeric sequences are copied from sister chromatids or other telomeric templates (Figure 1). Hallmarks of ALT-positive cells include highly heterogeneous telomere lengths, extrachromosomal telomeric DNA circles (C-circles), and the presence of ALT-associated promyelocytic leukemia (PML) nuclear bodies (APBs), which serve as hubs for telomere recombination and repair [5]. ALT activation is strongly correlated with loss-of-function mutations in chromatin remodeling factors such as ATRX and DAXX [6,7,8], and often coexists with heightened replication stress and DNA damage signaling. From a clinical standpoint, ALT has traditionally been considered an adverse biomarker [9]. In aggressive cancers such as osteosarcoma, pancreatic neuroendocrine tumors, and various high-grade sarcomas, ALT positivity is associated with genomic instability [7,10,11], therapy resistance, and poor patient survival. This has led to a general perception of ALT as a marker of aggressive tumor biology. Yet, paradoxically, emerging evidence reveals that ALT may confer a survival advantage in specific tumor contexts. Among the molecular subtypes of GBM, ALT activation is predominantly found in the mesenchymal and proneural types, while other subtypes may retain telomerase activity (TA) [12]. In glioblastoma multiforme (GBM) and chondrosarcoma—both of which are historically “immune-cold” tumors with limited T-cell infiltration, poor immunogenicity, and restricted therapeutic responsiveness—ALT positivity has been associated with improved patient outcomes. This observation challenges the conventional view of ALT as uniformly detrimental and raises intriguing questions about tumor-type-specific interactions between telomere biology, chromatin state, DNA repair stress, and the tumor microenvironment [13,14,15].

Furthermore, a major source of genomic instability in ALT is not solely replication stress but also arises from telomere uncapping and end-to-end chromosome fusions, which generate breakage–fusion–bridge cycles and further amplify chromosomal rearrangements and genomic instability characteristic of ALT-positive tumors. Understanding why ALT confers poor prognosis in most cancers [16,17] but a favorable prognosis in GBM and chondrosarcoma [14,18,19,20] is not only biologically significant but may also inform precision oncology strategies. By dissecting this paradox, we may uncover new insights into the interplay between telomere maintenance mechanisms and tumor evolution, as well as identify novel therapeutic vulnerabilities.

## 2. ALT and Poor Prognosis in Most Cancers

### 2.1. Genomic Instability as a Double-Edged Sword

The defining feature of ALT is its reliance on homologous recombination to elongate telomeres [21]. Unlike telomerase, which adds repeats in a relatively controlled manner, ALT operates through template switching and DNA repair pathways. This process, while ensuring telomere maintenance, comes at the cost of chronic genomic instability [7,21].

ALT-positive cells exhibit highly heterogeneous telomere lengths—ranging from extremely short to abnormally long—creating a population of highly heterogeneous telomeres prone to replication stress and recombination-mediated instability [7,22,23,24,25,26]. Such instability promotes the formation of anaphase bridges, dicentric chromosomes, and extensive chromosomal rearrangements. These aberrations not only fuel tumor evolution by generating genetic diversity but also compromise genomic integrity, providing fertile ground for aggressive behavior and therapeutic resistance [4,15,27].

### 2.2. ALT and Tumor Progression

The genomic instability associated with ALT does not occur in isolation; it actively shapes tumor progression. ALT-positive tumors often display: Enhanced invasiveness and metastatic potential, driven by chromosomal rearrangements that activate oncogenes or inactivate tumor suppressors [25,26]. Resistance to DNA-damaging therapies, such as radiation and alkylating chemotherapy, is due to heightened activation of DNA damage response pathways. Activation of recombination-based repair networks, particularly those mediated by RAD51 and the MRN complex, allows tumor cells to tolerate otherwise lethal DNA lesions. Consequently, ALT provides not only a means of telomere maintenance but also a platform for adaptive evolution, enabling cancer cells to survive in hostile microenvironments and under therapeutic stress [5,10,11,25,28,29].

### 2.3. Clinical Observations Across Cancer Types

Accumulating evidence has established ALT as a negative prognostic biomarker in several malignancies. Osteosarcoma shows a strong association between ALT positivity and poor outcomes, independent of tumor stage, metastasis status, or treatment modality, with patients typically experiencing reduced progression-free and overall survival compared to their ALT-negative counterparts. In pancreatic neuroendocrine tumors (PanNETs), ALT correlates with higher grade, metastatic disease, and reduced survival, reflecting the aggressive biology driven by ATRX/DAXX loss of function [7,8]. Similarly, in soft-tissue sarcomas, several subtypes demonstrate worse clinical outcomes when ALT is present, reinforcing its role as a marker of aggressive tumor biology. These findings suggest that in most cancer settings, ALT functions not as a benign alternative to telomerase but as a driver of malignant progression, enabling continued proliferation while simultaneously destabilizing the genome [9,13,30,31,32].

### 2.4. Summary

Taken together, the combination of ongoing recombination-driven instability, enhanced DNA repair capacity and pathway plasticity, and observed clinical correlations position ALT as a hallmark of poor prognosis in many cancers. ALT represents a molecular paradox—serving as a mechanism that counteracts telomere erosion while simultaneously promoting the genomic instability that drives malignant progression [33].

## 3. Favorable Prognosis of ALT in GBM and Chondrosarcoma

**Figure 2:**
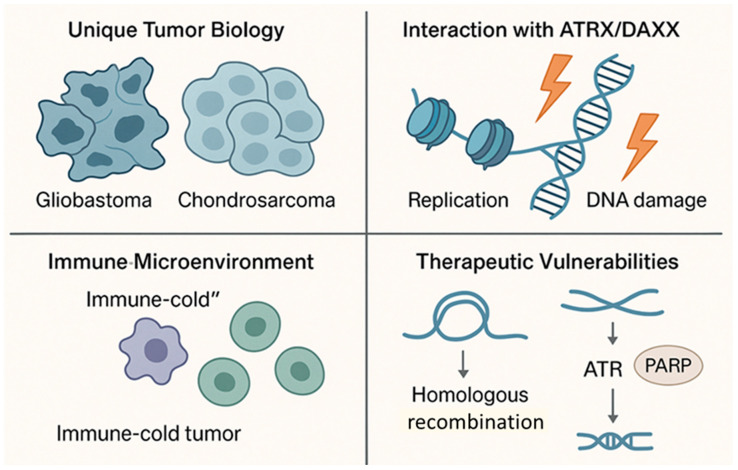
**ALT-Associated Favorable Prognosis and Distinct Tumor Biology in GBM and Chondrosarcoma.**

Although ALT is generally associated with poor outcomes [9,34], a striking exception emerges in glioblastoma (GBM) [35] and chondrosarcoma, two tumor types historically categorized as immune-cold malignancies with limited treatment responsiveness. In these contexts, ALT positivity correlates with improved patient survival, suggesting a context-dependent dual role of ALT (Figure 2) [13,36].

This scheme illustrates how ALT influences tumor behavior and therapeutic response in glioblastoma (GBM) and chondrosarcoma. ALT-positive tumors display a unique tumor biology characterized by relatively slow proliferation, persistent replication stress, and a reliance on homologous recombination for telomere maintenance. Loss-of-function mutations in ATRX or DAXX impair chromatin remodeling and increase DNA-damage sensitivity, creating a dependency on the ATR/PARP signaling axis. Although these tumors are generally immune-cold—showing limited immune-cell infiltration and low basal immune activation—ALT-mediated genomic instability may paradoxically trigger localized immune stimulation. Together, these features define a molecular context in which ALT positivity contributes to improved clinical outcomes and exposes selective therapeutic vulnerabilities that can be exploited through ATR or PARP inhibition.

### 3.1. Unique Tumor Biology

Glioblastoma [35] and chondrosarcoma exhibit distinct biological features that may help explain the paradoxical benefits of ALT. Glioblastoma is characterized by highly infiltrative growth, intratumoral heterogeneity, and metabolic adaptation, yet its proliferative index is often heterogeneous, with subpopulations cycling slowly. Chondrosarcoma [14], especially the conventional subtype, is typically slow-growing, with extensive extracellular matrix deposition that restricts vascular supply and limits rapid cell division. In such biological contexts, ALT may impose a fitness cost. Because recombination-based telomere maintenance is inefficient and associated with replication stress, ALT-positive cells may experience delayed S-phase progression, increased checkpoint activation, and energy-intensive DNA repair processes. This replicative burden could effectively restrain net tumor growth compared to telomerase-positive counterparts, inadvertently benefiting patient prognosis [35].

### 3.2. Interaction with ATRX/DAXX Mutations

A central mechanistic link between ALT and tumor biology lies in ATRX and DAXX, chromatin remodelers critical for the deposition of histone variant H3.3 at telomeric and pericentromeric regions. Loss of ATRX/DAXX [8,36] is a near-universal hallmark of ALT activation. Without ATRX/DAXX [7,37], chromatin becomes less compact and more vulnerable to replication stress. In GBM [38], ATRX deficiency synergizes with ALT to exacerbate DNA replication stress, making tumor cells more susceptible to spontaneous DNA breaks and reduced proliferative fitness. This vulnerability may explain why ALT-positive GBMs do not achieve the same level of aggressiveness as telomerase-positive tumors, despite their genomic instability [16,31]. In this sense, ATRX/DAXX mutations [38,39] convert ALT into a double liability: while they enable telomere maintenance, they also amplify the detrimental effects of replication stress and chromatin fragility [22,40,41,42]. It is noteworthy, however, that this synergistic interaction between ATRX deficiency and ALT activation observed in GBM does not appear uniformly across all ALT-positive tumors. Although many poor-prognosis ALT cancers also harbor ATRX/DAXX mutations, their biological contexts differ substantially. In GBM, the high baseline levels of replication stress and oxidative DNA damage likely amplify the deleterious effects of ATRX loss, leading to reduced proliferative fitness. In contrast, in more proliferative or metabolically adaptable tumors, compensatory DNA repair and checkpoint pathways may buffer this stress, preventing the same synergistic fragility observed in GBM. This context dependence underscores that ATRX/DAXX loss is not intrinsically protective or detrimental, but its outcome is shaped by the tumor’s replication dynamics and genomic environment [37,43,44].

### 3.3. Immune Microenvironment

The immune landscape is another critical determinant of ALT’s prognostic impact.

Immune-cold tumors, such as GBM and chondrosarcoma, are characterized by low T-cell infiltration, absence of strong antigen presentation, and resistance to checkpoint blockade therapies. ALT-mediated genomic instability, however, produces extrachromosomal telomeric DNA, cytosolic DNA fragments, and potential neoantigens through aberrant recombination events [45,46,47]. These byproducts may activate innate immune pathways such as cGAS–STING signaling [45,46,47], triggering interferon responses and localized immune surveillance. Thus, in the otherwise quiescent immune environment of GBM and chondrosarcoma, ALT may paradoxically increase immunogenicity just enough to slow tumor progression, without triggering full-scale immune evasion mechanisms that are typical of immune-hot tumors.

### 3.4. Therapeutic Vulnerabilities

Another layer of explanation comes from the therapeutic vulnerabilities created by ALT dependence. ALT-positive cells rely heavily on homologous recombination repair and ATR-dependent DNA damage signaling to survive continuous telomeric stress [8,48]. This dependency exposes them to synthetic lethality when DNA damage response (DDR) pathways are pharmacologically targeted. For example, ATR inhibitors can collapse replication forks in ALT+ cells, leading to catastrophic telomere dysfunction. PARP inhibitors further exacerbate DNA repair deficiencies, particularly in ATRX-deficient, ALT-positive settings. In GBM and chondrosarcoma—tumors with historically poor responsiveness to conventional chemotherapy—such ALT-specific vulnerabilities may help explain why ALT-positive patients exhibit relatively better survival when compared with ALT-negative cases [8,40,41,49,50].

### 3.5. Summary

The favorable prognosis observed in ALT-positive glioblastoma (GBM) and chondrosarcoma can be attributed to several converging biological factors. In metabolically constrained or replication-stressed tumors, recombination-based telomere maintenance imposes a proliferative limitation, while ATRX/DAXX loss of function further exacerbates genomic instability and reduces growth potential. Within immune-cold microenvironments, ALT-mediated genomic instability may paradoxically enhance local immunogenicity, collectively contributing to slower tumor progression and improved clinical outcomes. “Although clinical data specifically in ALT-positive GBM or chondrosarcoma remain limited, preclinical studies have shown strong ATR dependency in ALT and ATRX-deficient settings [40,41,48]. Several ATR inhibitors, including ceralasertib and berzosertib, are currently being evaluated in early-phase clinical trials for DDR-defective gliomas and sarcomas”.

Together, these biological, genetic, and microenvironmental influences shift ALT from a driver of aggressiveness in most cancers to a relative weakness in GBM and chondrosarcoma, thereby inverting its typical prognostic role.

## 4. A Context-Dependent Model

**Figure 3:**
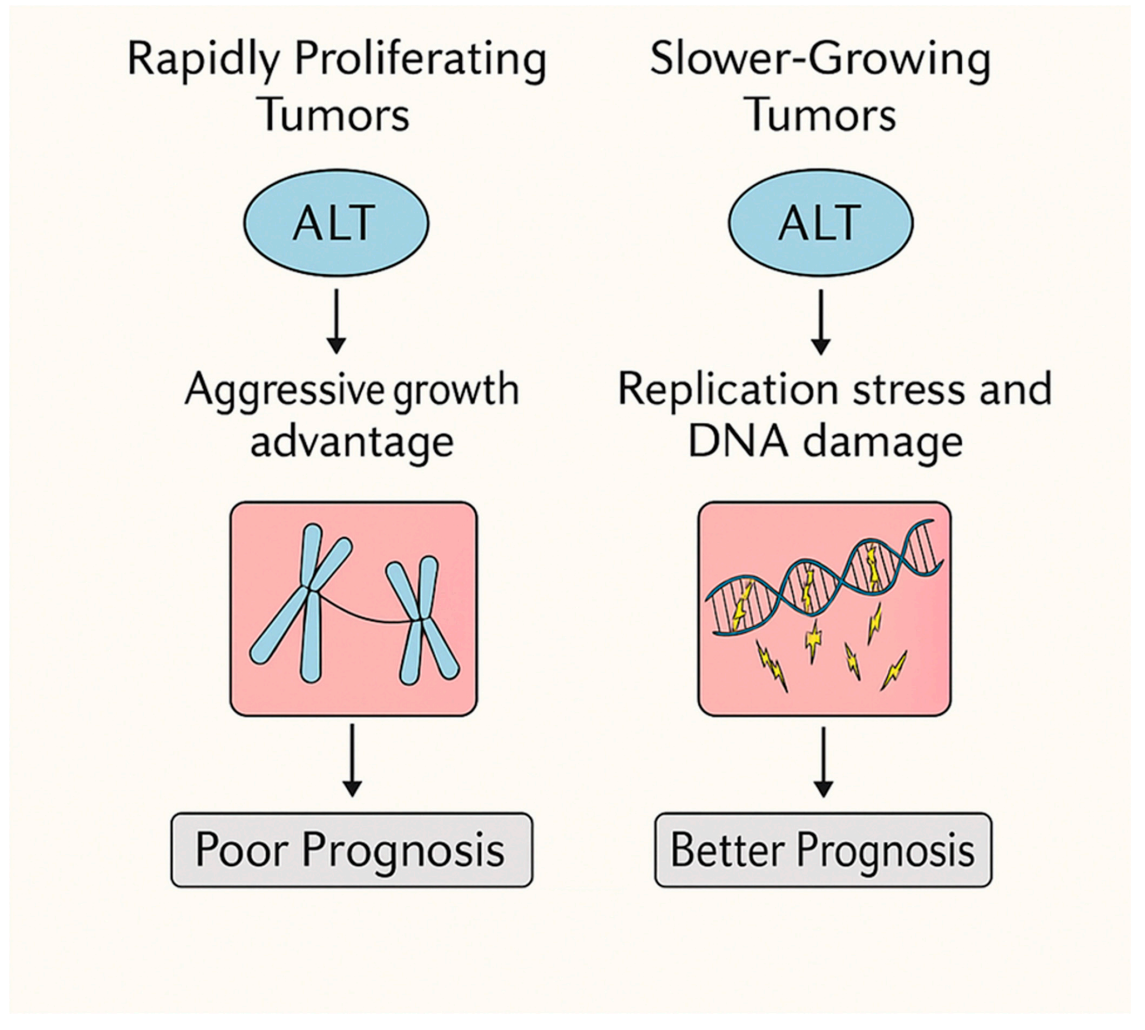
**Context-Dependent Prognostic Impact of ALT in Cancer.** This scheme illustrates how the clinical outcomes of Alternative Lengthening of Telomeres (ALT) depend on the proliferative and genomic context of the tumor. In rapidly proliferating tumors, ALT confers a transient growth advantage but simultaneously drives chromosomal instability, contributing to poor prognosis. In contrast, replication stress and the accumulation of DNA damage impose a proliferative constraint on ALT-positive tumors, leading to slower tumor growth and improved prognosis. ALT therefore functions as a double-edged sword—its prognostic impact determined by whether replication stress predominates over proliferative advantage within the tumor’s biological context.

The paradoxical role of alternative lengthening of telomeres (ALT) across cancer types underscores the importance of tumor context in shaping the biological and clinical consequences of telomere maintenance mechanisms. ALT cannot be viewed as uniformly detrimental or beneficial; rather, its impact emerges from the dynamic interplay between tumor proliferation rate, chromatin architecture, immune microenvironment, and therapeutic vulnerabilities (Figure 3). The multifactorial determinants shaping ALT activation and its phenotypic outcomes across tumor types are summarized in Table 1.

### 4.1. ALT as a Driver of Aggressiveness in Rapidly Proliferating Tumors

In highly proliferative cancers, the need for robust and efficient telomere elongation is paramount. Telomerase-negative tumors that adopt ALT can achieve continued immortality by engaging recombination-based mechanisms. In this setting, Adaptive Advantage: ALT sustains telomere length despite high mitotic demand, allowing cancer cells to bypass replicative senescence; Oncogenic Diversity: Ongoing telomeric recombination generates chromosomal rearrangements and structural variations, which may activate oncogenes or disable tumor suppressors. The combination of cellular immortality and genomic plasticity has significant clinical implications, as it accelerates disease progression, promotes metastasis, and contributes to therapeutic resistance by enabling tumor cells to adapt to genomic instability and environmental stress.

Thus, in fast-growing malignancies such as osteosarcoma or pancreatic neuroendocrine tumors, ALT functions as a growth enabler whose benefits outweigh its costs, leading to poor prognosis [13,17].

### 4.2. ALT as a Fitness Burden in Slow-Growing, Immune-Cold Tumors

In contrast, in intrinsically slow-growing, immune-cold tumors such as glioblastoma and chondrosarcoma, the ALT mechanism manifests as a fitness burden rather than a proliferative advantage.

ALT-driven replication stress increases reliance on homologous recombination and DNA damage signaling. The pronounced telomeric fragility observed in ALT-positive tumors reflects persistent replication stress and contributes to overall genomic instability [2,7,11].

In the context of low proliferative drive, replication stress and metabolic constraints impose a burden that limits overall tumor growth. In immune-cold tumors with minimal baseline immune activity, the low-level genomic instability induced by ALT may paradoxically generate neoantigens or cytosolic DNA fragments that activate innate immune pathways. Instead of promoting immune evasion as in immune-hot tumors, these signals may provide sufficient immunogenicity to slow tumor progression.

Chromatin remodeling defects resulting from ATRX/DAXX loss of function, a common co-occurrence with ALT, further sensitize these tumors to replication stress and enhance their intrinsic fragility. In this context, ALT becomes less of an advantage and more of a liability, shifting the balance toward reduced aggressiveness and a relatively improved prognosis [9,14].

While ALT-positive tumors generally display genomic instability, in immune-cold and slow-growing contexts such as glioblastoma or chondrosarcoma, these alterations may exert limited evolutionary pressure—a hypothesis that requires further experimental validation.

### 4.3. ALT as a Double-Edged Sword

Taken together, ALT exemplifies a double-edged sword in cancer biology. In rapidly proliferating, telomerase-negative cancers, ALT sustains cellular immortality and accelerates genomic evolution, ultimately leading to poor prognosis. In contrast, in slower-growing, immune-cold tumors, ALT amplifies DNA damage and replication stress, reducing tumor fitness and resulting in a relatively better prognosis.

This duality highlights how the same molecular mechanism can generate opposite clinical outcomes depending on the surrounding biological landscape [15].

### 4.4. Broader Implications

This context-dependent framework carries important implications: Prognostic Stratification: ALT status should not be interpreted uniformly across cancers; its meaning depends on tumor type, proliferation rate, and immune contexture. Therapeutic Targeting: ALT-related vulnerabilities (e.g., reliance on ATR, PARP, or homologous recombination) may be exploitable in ALT-positive cancers, but therapeutic efficacy will vary depending on whether ALT acts as a driver or a burden. The paradox of ALT suggests that telomere maintenance mechanisms are not merely survival tools but also represent evolutionary trade-offs, with distinct costs and benefits shaped by the tumor ecosystem [51]. This synergism between ALT-induced DNA damage and checkpoint inhibition (e.g., ATR blockade) likely exists across both poor- and better-prognosis ALT tumors, but its biological impact is context dependent. In slow-growing and metabolically constrained tumors such as GBM and chondrosarcoma, chronic replication stress places the DNA damage response near its tolerance threshold; thus, additional ATR inhibition precipitates replication collapse and cell death, amplifying the therapeutic effect. Conversely, in rapidly proliferating ALT tumors with more efficient repair and checkpoint compensation, this synergy is less pronounced. Likewise, the interaction between p53 inactivation and ALT-driven DNA damage under ATRX/DAXX deficiency may either accelerate genomic instability or, when p53 remains intact, restrain tumor proliferation through cell-cycle arrest and apoptosis. These contrasts highlight that the consequences of ALT-related stress are dictated not by the presence of ATRX or p53 loss alone, but by the tumor’s broader replication, metabolic, and DNA repair landscape.

### 4.5. ALT, p53, and Tumor Suppressor Networks

Emerging evidence indicates that the interplay between ALT activation and the p53 tumor suppressor pathway exerts a profound influence on tumor evolution and therapeutic vulnerabilities. p53 is a central guardian of genomic integrity, activated by replication stress, telomere dysfunction, and DNA damage—all hallmarks of ALT-positive cells. Inactivation of p53 through mutation or deletion can enable ALT-driven cells to bypass senescence and apoptosis despite chronic telomeric stress, thereby promoting oncogenesis. Conversely, intact p53 signaling may constrain ALT-positive cells by enforcing cell-cycle arrest or apoptosis in response to persistent DNA damage and telomere instability. Recent studies suggest that p53 dysfunction may cooperate with ATRX/DAXX loss of function to accelerate genomic rearrangements and tumor progression in ALT-positive contexts, highlighting a potential synthetic vulnerability that could be therapeutically exploited [40,52,53].

### 4.6. Oncogenic and Tumor-Suppressive Alterations in ALT-Driven Cancers

The molecular landscape of ALT-positive tumors is shaped not only by telomere maintenance mechanisms but also by broader oncogenic and tumor-suppressive alterations [6,7]. Frequent co-occurrence of ALT activation with mutations in ATRX, DAXX, TP53, and RB1 underscores its role within a complex genomic network that modulates cell-cycle control, chromatin structure, and DNA repair. Oncogenic drivers such as MYC or RAS may amplify the proliferative pressure in ALT-positive cells, exacerbating replication stress and fueling further chromosomal instability. At the same time, loss of tumor suppressors (e.g., p53 or RB) dismantles checkpoints that would otherwise limit ALT-driven proliferation. The combined effect of these alterations enhances tumor adaptability but also exposes unique vulnerabilities, suggesting that ALT biology must be understood within the broader mutational context of cancer genomes [54].

### 4.7. Oxidative Stress as a Modulator of ALT Dynamics

Oxidative stress represents another critical factor influencing the biology of ALT-positive tumors. Elevated levels of reactive oxygen species (ROS) can accelerate telomere shortening, promote DNA double-strand breaks, and intensify replication stress—all of which potentiate ALT activation. Conversely, ALT-mediated genomic instability itself may amplify oxidative damage, establishing a self-reinforcing feedback loop. Intriguingly, ROS-induced DNA lesions can stimulate recombination-based telomere synthesis, potentially enhancing ALT activity under conditions of metabolic stress [55,56,57]. The interplay between oxidative stress and telomere maintenance may therefore determine not only the efficiency of ALT activation but also the sensitivity of ALT-positive tumors to redox-modulating therapies [58,59].

### 4.8. Epigenetic Regulation, Metabolism, and the Tumor Microenvironment

Finally, epigenetic regulation and the tumor microenvironment exert powerful effects on ALT biology and clinical outcomes. Epigenetic remodeling—including histone modifications, chromatin accessibility, and DNA methylation—can modulate the recombination machinery at telomeres, influence the transcription of telomere-associated factors, and even dictate whether ALT activation is sustainable. For instance, loss of heterochromatin marks at telomeres may facilitate the recruitment of recombination complexes, while metabolic cues from the tumor microenvironment can shape chromatin states that either promote or restrain ALT [39,43,60]. Moreover, the metabolic landscape—such as hypoxia, nutrient availability, and mitochondrial function—intersects with ALT activity by altering DNA repair capacity and replication stress responses [47,61,62]. Together, these epigenetic and microenvironmental influences underscore that ALT should be viewed not merely as a telomere maintenance mechanism, but as an integrated process shaped by chromatin dynamics, metabolic state, and the ecological context of the tumor.

## 5. Clinical Implications

### 5.1. Prognostic Stratification

**Figure 4:**
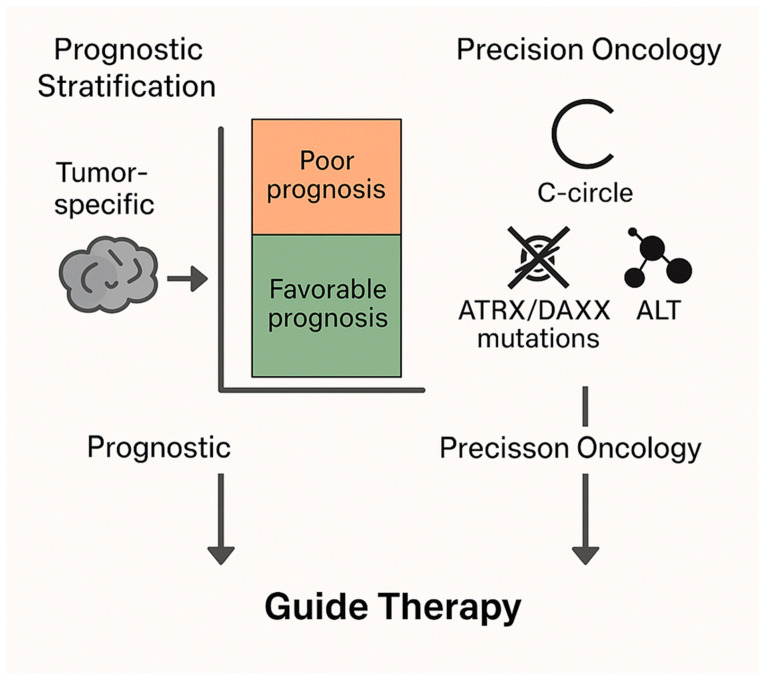
**Clinical Utility of ALT for Prognosis and Therapy Guidance.** ALT serves as a tumor-specific biomarker with dual roles: It enables prognostic stratification, predicting favorable or poor outcomes depending on cancer type. It supports precision oncology, where biomarkers such as C-circles and ATRX/DAXX mutations guide targeted therapies. ALT status can help personalize treatment decisions in cancer care.

The recognition that ALT does not uniformly predict poor outcomes, but rather exerts context-dependent effects, calls for a paradigm shift in how telomere biology is integrated into oncology practice. In highly proliferative cancers such as osteosarcoma or pancreatic neuroendocrine tumors, ALT positivity should still be considered a marker of aggressive disease and unfavorable prognosis. However, in slower-growing, immune-cold tumors such as glioblastoma (GBM) and chondrosarcoma, ALT status may actually serve as a favorable prognostic indicator. This distinction emphasizes the need for tumor-type-specific interpretation of ALT biomarkers rather than a “one-size-fits-all” approach (Figure 4).

### 5.2. Therapeutic Targeting

ALT-positive cancers exhibit unique molecular dependencies that create therapeutic vulnerabilities. Because ALT relies on homologous recombination-mediated DNA repair and ATR-dependent replication stress responses, inhibitors targeting these pathways have shown therapeutic potential. ATR inhibition can destabilize ALT-dependent replication forks, causing replication collapse and severe telomere dysfunction that ultimately compromises ALT-mediated telomere maintenance. PARP inhibitors disrupt single-strand break repair and trap PARP–DNA complexes, leading to replication fork collapse and double-strand break accumulation; in ALT-positive cells, this amplifies replication stress and induces synthetic lethality. PARP inhibitors, already approved for the treatment of homologous recombination-deficient cancers, may exhibit enhanced therapeutic efficacy in ALT-positive tumors, especially those harboring ATRX/DAXX loss of function, due to increased reliance on recombination-mediated telomere maintenance [63,64,65,66,67,68,69,70]. DNA damage–inducing therapies (radiation, alkylating agents) may exert greater long-term effects on ALT+ GBM or chondrosarcoma due to their compromised ability to maintain genomic stability. Thus, ALT+ tumors represent a distinct therapeutic subset in which exploiting DNA repair vulnerabilities could transform patient outcomes, especially in cancers where conventional therapies have limited success.

### 5.3. Precision Oncology

Integration of ALT-specific biomarkers into clinical workflows is a promising frontier. Established assays, such as C-circle assays (extrachromosomal telomeric DNA detection), immunofluorescence for ALT-associated PML bodies (APBs), and molecular profiling of ATRX/DAXX mutations, can be combined with genomic and transcriptomic profiling to identify ALT-positive tumors with high accuracy. Incorporating these markers into clinical diagnostics would enable: More precise prognostication, by distinguishing aggressive ALT+ tumors from those in which ALT predicts better survival. Personalized therapeutic decision-making, including selection of DDR-targeting agents or clinical trials tailored to ALT biology. Dynamic monitoring of treatment response, since ALT activity can fluctuate under therapeutic stress. In the era of precision oncology, ALT biology provides both a prognostic biomarker and a therapeutic target, bridging fundamental telomere biology with actionable clinical strategies [71,72,73,74,75].

## 6. Conclusions

ALT acts as a double-edged sword in cancer biology: it drives poor prognosis in rapidly proliferating, telomerase-negative tumors, yet can confer a survival advantage in slower-growing, immune-cold cancers. Recognizing this context-dependent duality establishes ALT as both a prognostic biomarker and a therapeutic target, with potential to refine patient stratification and guide precision oncology strategies.

## Figures and Tables

**Table 1:** **Summary of the main parameters influencing the adoption and phenotypic outcome of the ALT (Alternative Lengthening of Telomeres) pathway across tumor contexts.**

Parameter	Functional Role in ALT Activation	Effect on Tumor Phenotype	Interaction with Other Factors
Level of DNA Damage	Persistent replication stress and telomeric DNA breaks initiate homologous recombination-based telomere elongation.	High levels favor ALT activation but can impair proliferative fitness when excessive.	Synergizes with ATRX/DAXX loss and impaired checkpoint signaling.
ATRX/DAXX Mutations	Disrupt chromatin remodeling at telomeric regions, facilitating recombination-mediated telomere synthesis.	Promote genomic instability; context-dependent effect (protective vs. deleterious).	Strongly linked to replication stress and p53 status.
Checkpoint Mutations (ATR/ATM Pathways)	Impair DNA damage sensing and repair coordination.	Can enhance ALT activation under chronic stress; may also promote cell death if damage exceeds tolerance.	Modulate replication stress response in combination with ATRX/DAXX loss.
Proliferation Rate	Determines cellular tolerance to replication stress and repair efficiency.	Low proliferation favors stable ALT activation (e.g., GBM, chondrosarcoma), while high proliferation supports telomerase-based maintenance.	Influenced by metabolic capacity and cell cycle control.
p53 Status (*Secondary factor*)	Controls cell-cycle arrest and apoptosis in response to DNA damage.	p53 loss allows accumulation of DNA lesions; intact p53 suppresses uncontrolled ALT proliferation.	Interacts with ATRX/DAXX and checkpoint pathways.
Epigenetic Regulation (*Secondary factor*)	Modifies chromatin accessibility at telomeric/subtelomeric loci.	Aberrant histone modification or DNA methylation can trigger or stabilize ALT activation.	Affects ATRX/DAXX recruitment and replication timing.
Metabolic State (*Secondary factor*)	Alters nucleotide pool balance and redox state, influencing DNA replication fidelity.	Metabolic stress enhances ALT vulnerability; metabolic adaptation supports ALT persistence.	Links cellular energy metabolism to replication stress resilience.

## Data Availability

Not applicable.
